# Efficacy of acupuncture or moxibustion in treating senile insomnia compared with a control group: A systematic review and meta-analysis

**DOI:** 10.1097/MD.0000000000034842

**Published:** 2023-10-20

**Authors:** Wenjiao Hu, Hao Zhou, Yue Zeng, Qian Zeng, Zubo Huang, Chao Wang

**Affiliations:** a Chengdu University of Traditional Chinese Medicine, Chengdu, China; b Sichuan Integrative Medicine Hospital, Chengdu, China.

**Keywords:** acupuncture, insomnia, meta-analysis, moxibustion, senile

## Abstract

**Introduction:**

This systematic review and meta-analysis aimed to assess the efficacy of acupuncture or moxibustion therapy in senile insomnia patients.

**Methods:**

A comprehensive literature search was conducted using 7 electronic databases to identify randomized controlled trials reported on the use of acupuncture or moxibustion therapy in insomnia. The time frame was set from database establishment to March 11, 2023. The RevMan (version 5.3) and STATA (version 17.0) software were used to evaluate the quality of the included randomized controlled trials and perform a meta-analysis. The methodological quality of the included studies was assessed using the Cochrane risk-of-bias tool. Subgroup analysis was performed based on different intervention methods. The I^2^ statistic was used to assess heterogeneity among studies.

**Results:**

A total of 20 studies conducted between 2007 and 2022 were included, involving 1677 patients with senile insomnia. In terms of efficacy, acupuncture or moxibustion alone was significantly better than western drugs (RR = 1.12; 95% CI, 1.06–1.20), acupuncture combined with drugs was better than drugs alone (RR = 1.20; 95% CI, 1.12–1.29), and acupuncture combined with cognitive behavior therapy intervention (CBT-I) was significantly better than CBT-I alone (RR = 1.52; 95% CI, 1.07–2.17). In terms of Pittsburgh Sleep Quality Index scores, acupuncture or moxibustion alone was more effective than western drugs (MD = −1.82; 95% CI, −2.37 to −1.26), acupuncture combined with drugs was more effective than drugs alone (MD = −3.10; 95% CI, −4.25 to −1.95), and acupuncture was significantly more effective than sham acupuncture (MD = −4.18; 95% CI, −5.85 to −2.51) and psychological intervention (MD = −3.54; 95% CI, −4.33 to −2.75) in improving sleep quality.

**Conclusions:**

This meta-analysis revealed that acupuncture or moxibustion alone or combination with other therapies(drugs, CBT-I or psychological intervention) has high clinical efficacy and can improve the sleep quality of patients with senile insomnia. However, further well-designed studies are warranted to verify these findings.

## 1. Introduction

Insomnia is a common sleep disorder in modern society.^[1]^ It is mainly characterized by difficulty initiating or maintaining sleep or both. The prevalence of insomnia increases with age.^[2]^ Studies have reported that 50% of the elderly population has difficulty falling or remaining asleep.^[3,4]^ Insomnia can be classified as primary or secondary based on its cause. Primary insomnia refers to the presence of symptoms of insomnia that cannot be attributed to an existing condition, whereas secondary insomnia may be caused by somatic organic diseases, alcohol, caffeine, drugs, anxiety, and depression. The primary cause of senile insomnia is aging, which leads to degeneration of the central nervous system, resulting in a disturbed sleep rhythm at night. Chronic insomnia greatly affects function in the daytime, thereby impairing memory and concentration and increasing the incidence of many common chronic diseases and the risk of neuropsychiatric comorbidities such as anxiety or depression.^[5–7]^ Insomnia is primarily treated with CBT-I for insomnia) and medications such as BZs, BZRAs, antidepressants, antipsychotics, antihistamines, plant treatment substances, and melatonin.^[8]^ Benzodiazepines are widely used to treat insomnia; however, their efficacy is limited owing to drug resistance, addiction, withdrawal, and other adverse reactions.

Acupuncture and moxibustion are part of external treatment and have been widely used to treat senile insomnia in traditional Chinese medicine. Studies have reported that the use of acupuncture and moxibustion in the treatment of insomnia has the advantages of accurate efficacy, simple operation, and fewer adverse reactions.^[7,9–25]^ However, the efficacy of these approaches remains elusive owing to the insufficient number of high-quality, well-designed randomized controlled trials. This systematic review and meta-analysis demonstrated the efficacy of acupuncture and moxibustion in the treatment of senile insomnia.

## 2. Methods

### 2.1. Trial registration

This review was registered on PROSPERO (CRD42023403274; https://www.crd.york.ac.uk/prospero/), without amendments between the registration and the final article. The study was conducted in accordance with the PRISMA guidelines but without a published protocol.

### 2.2. Literature search

A literature search was performed in the following English and Chinese databases from the date of database establishment to March 11, 2023: PubMed, Embase, Cochrane Library, China Knowledge Resource Integrated Database (CNKI), Chongqing VIP Information, Wanfang Database, and Chinese Biomedical Literature Database. The search was conducted using subject words plus free words. English search terms based on the Medical Subject Headings thesaurus were “insomnia” OR “sleeplessness” OR “sleep disorder” and “senile” or “elder” and “acupuncture” OR “electroacupuncture” OR “moxibustion” and “random control” and “randomized controlled trial” and “RCT.” The search strategy for Chinese databases was adjusted for Chinese medical terms and their usage in the literature. The following terms were searched in the Chinese databases: SHI MIAN (“insomnia”), BU MEI (“insomnia”), SHUI MIAN ZHANG AI (“insomnia”), LAO NIAN (“senile”), ZHEN JIU (“acupuncture”), ZHEN CI (“acupuncture”), DIAN ZHEN (“electroacupuncture”), JIU FA (moxibustion), and SUI JI DUI ZHAO (randomized controlled). Search terms from the Medical Subject Headings were “Dyssomnias” and “Acupuncture Therapy.” (Research strategy was listed in supplemental files 1, http://links.lww.com/MD/J647)We examined the reference lists of relevant articles to identify the citations not captured by the electronic searches. The corresponding authors were contacted for missing information.

### 2.3. Inclusion and exclusion criteria

Randomized controlled trials (RCTs) assessing the efficacy of acupuncture or moxibustion in the treatment of senile insomnia were included based on the following criteria: types of participants: Patients (age, ≥60 years) with senile insomnia irrespective of race and sex; types of studies: RCTs and studies in which blinding and allocation concealment were not limited; and types of interventions: The experimental group was treated with acupuncture, electroacupuncture, or moxibustion alone or acupuncture combined with other therapies, whereas the control group was treated with sham acupuncture alone, placebo, or other therapies used in the treatment group; types of outcomes: Primary outcomes included the rate of improvement and the Pittsburgh Sleep Quality Index (PSQI) score.^[26]^ The PSQI consists of 7 components: subjective sleep quality, sleep latency, sleep duration, habitual sleep efficiency, sleep disturbances, use of sleeping medications, and daytime dysfunction. The PSQI is a widely used measure of sleep quality that is more global in nature than other measures. Only Chinese and English articles that met the above mentioned inclusion criteria were included.

The following articles were excluded: quasi-randomized RCTs and non-randomized trials, duplicate publications, and studies with unavailable full text or missing data.

Two reviewers (Wenjiao Hu and Hao Zhou) independently screened the titles and abstracts of the selected studies based on presupposed inclusion and exclusion criteria and assessed the full text of potentially eligible studies. If case of disagreement, another reviewer Yue Zeng was consulted to resolve any uncertainties regarding study inclusion.

### 2.4. Data extraction

Two authors Wenjiao Hu and Hao Zhou extracted data of articles. The third author Qian Zeng was consulted, if the 2 authors disagreed. The following data were extracted in a predefined data collection form: first author, year of publication, language, sample size, demographic data of participants, baseline characteristics of patients, diagnostic criteria, inclusion and exclusion criteria for participants, experimental and control interventions, course of treatment, frequency, location of the study, outcomes, and safety.

### 2.5. Quality assessment

The Cochrane risk-of-bias tool for RCTs was used to assess potential bias in the included studies.^[27]^ The risk-of-bias tool consists of 7 domains as follows: random sequence generation, allocation concealment, blinding of participants and personnel, blinding of outcome assessment, incomplete outcome data, selective reporting, and other bias (we assessed trials with no reported monitoring of self-acupressure procedures with a high risk of compliance bias). Each RCT was categorized as having a low, high, or unclear risk of bias in each domain.

### 2.6. Data synthesis and statistical analysis

The Review Manager (RevMan) (version 5.3) (Copenhagen, Denmark: The Nordic Cochrane Centre, The Cochrane Collaboration, 2014) and STATA (version 17.0) software were used for qualitative analysis of the included studies. Combination of effects: Continuous outcomes (PSQI) were evaluated based on weighted mean differences (WMDs), and dichotomous outcomes (rate of improvement) were assessed based on risk ratios (RRs). The 95% confidence interval (95% CI) was evaluated for all effect sizes, with a 95% CI excluding the point of no effect indicating statistical significance. Heterogeneity test: The Q-test was performed to assess heterogeneity among studies. If *P* values were >.10, the results of multiple similar studies were considered homogenous. If *P* values were >.10 and I^2^ values were ≥ 0 and ≤ 50%, a fixed effects model was used for integrative analysis of studies. If *P* values were ≤.10 or I^2^ values were > 50%, the results of multiple similar studies were considered heterogeneous, and sensitivity analysis was performed. The articles were removed sequentially to observe changes in heterogeneity, WMDs, and RRs. If heterogeneity was altered after the removal of an article, it was considered the source of heterogeneity, and the underlying reason was analyzed. If heterogeneity remained unaltered, indicating stable results, a random effects model was used for a more conservative evaluation of the intervening effects. Subgroup analyses were performed based on acupuncture methods (single acupuncture vs single moxibustion vs acupuncture combined with other therapies). Given that ≥ 10 studies were included in the meta-analysis, Egger test was performed to assess publication bias, with a *P* value of <.05 indicating significance.^[28]^

### 2.7. Ethical review instructions

Although the specimens taken are human (or animal), this is a secondary analysis of the article and does not involve ethical issues.

## 3. Results

### 3.1. Study selection

**Figure 1.**
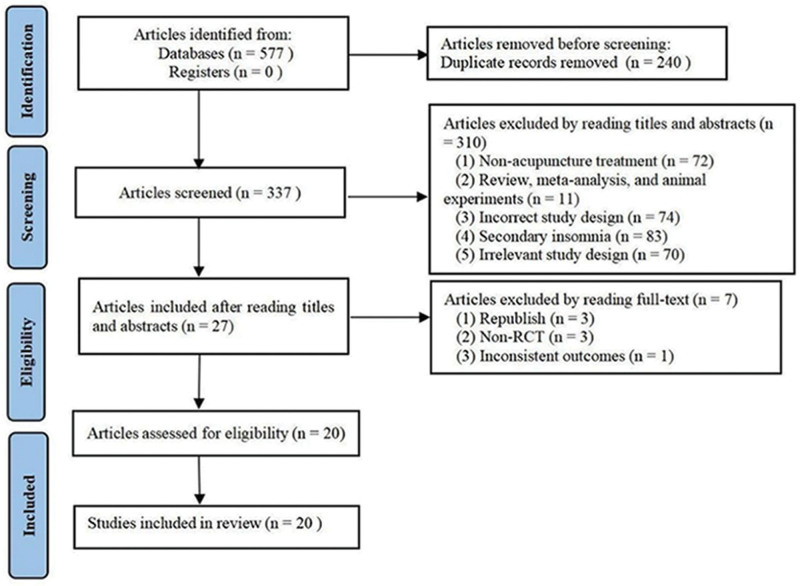
Flowchart of study selection.

A total of 577 articles were selected from the 7 databases; of which, 377 articles remained after the removal of duplicates. Of these 377 articles, most articles were excluded because they failed to meet the inclusion criteria. A total of 27 full-text articles were assessed to select those that met the eligibility criteria (Fig. 1). According to the inclusion and exclusion criteria, 20 articles were eventually included. The study inclusion process is illustrated in Figure 1.

### 3.2. Study characteristics

The baseline information of the 20 studies included in this systematic review and meta-analysis is summarized in Table 1. All the included RCTs originated in a single center in China and were published in Chinese. Of the 20 articles, 8 articles were RCTs that compared acupuncture or moxibustion alone with western drugs, 8 articles were RCTs that compared the combination of acupuncture or moxibustion plus drugs with drugs alone, 2 articles were RCTs that compared acupuncture with sham or placebo acupuncture, and 2 articles were RCTs that compared the combination of acupuncture plus cognitive behavioral intervention or psychological intervention with cognitive behavioral intervention or psychological intervention alone. Of the 1677 patients with insomnia, 866 were assigned to the experimental group and 811 were assigned to the control group, with 801 male patients and 876 female patients. All the trials reported non-significant differences in the baseline characteristics of patients. The rate of improvement was evaluated for examining therapeutic efficacy in 16 studies^[1–5,7,9,10]^; of which, 15 studies used the Pittsburgh Sleep Quality Index (PSQI).^[1,2,4–8,10–15,18]^ In addition, 2 studies evaluated the rate of improvement,^[1,10]^ and 4 studies evaluated PSQI scores during the follow-up period.^[1,7,8,10]^ The quality of the data was not high. Outcome details were listed in supplemental files 2, http://links.lww.com/MD/J648.

**Table 1:** Summary of basic characteristics of the included studies.

First author (yr)	Sample size (male/female)		Age in yr	Randomization method	Treatment	Control	Frequency	PSQI scores	Location	Blinding status	Treatment duration	Outcome	Safety
Yuxueping (2019)	28 (15/13)	28 (12/16)	71.3 ± 5.7/72.3 ± 4.8	Random number table	AC	Estazolam	Per day	14.82 ± 2.07/14.29 ± 2.67	Guangdong	NA	4 w	PSQI; effective rate	Not reported
Sunzhaoyuan (2010)	40 (22/18)	40 (19/21)	74.32 ± 6.604/74.48 ± 6.687	Not reported	AC	Estazolam	Per day	13.02 ± 2.89/13.15 ± 2.957	Tianjin	NA	4 w	PSQI; effective rate	Not reported
Xuewenxion (2017)	40 (17/23)	40 (18/22)	69.01 ± 2.15/69.02 ± 2.14	Not reported	AC	Estazolam	1 time, 2 d	NA	Jiangsu	NA	2 m	Effective rate	Not reported
Lishude (2021)	39 (17/22)	38 (20/18)	66.29 ± 4.96/65.62 ± 3.72	Random number table	AC	Estazolam	6 times per wk	6.36 ± 3.85/8.71 ± 3.08	Fujian	NA	4 w	PSQI; effective rate	Y
Tanzhiwei (2020)	47 (23/24)	47 (25/22)	70.03 ± 3.11/69.13 ± 6.84	Not reported	AC	Oryzanol and lorazepam	Per day	15.13 ± 3.08/15.06 ± 3.15	Hunan	Double blinding	4 w	PSQI; effective rate	Not reported
Wangxiaoqiu (2021)	30 (13/17)	30 (11/18)	69 ± 4/69 ± 5	Random number table	EA	Sham AC	3 times per wk	13.90 ± 2.86/14.24 ± 3.11	Jiangsu	Single blinding	4 w	PSQI	Y
Youxuyu (2019)	77 (34/43)	30 (12/18)	69.5 ± 2/70.5 ± 3	Random number table	AC	Estazolam	Per day	15.6 ± 2.025/15.7 ± 1.878	Zheijiang	NA	1 m	PSQI; effective rate	Not reported
Zhengyi (2020)	40 (18/22)	40 (17/21)	73.2 ± 3.3/72.2 ± 2.3	Not reported	Moxibustion	Diazepam	Per day	14.33 ± 2.43/14.12 ± 2.21	Guangxi	NA	20 d	PSQI	Not reported
Yangjingyi (2020)	40 (22/18)	40 (21/19)	62–72/61–71	Random number table	AC	Alprazolam	Per day	NA	Zheijiang	NA	4 w	Effective rate	Not reported
Zhanghanxiao(2022)	57 (25/32)	59 (28/31)	67.44	Not reported	Moxibustion	Estazolam/clonazepam/eszopiclone	Per day	8.95 ± 3.00/9.25 ± 2.83	Fujian	NA	20 d	PSQI; effective rate	Not reported
Chenjuan (2019)	51 (30/ 21)	49 (23/26)	63 ± 4/62 ± 3	Random number table	AC plus Chinese medicine	Chinese medicine	Per day	NA	Hebei	NA	20 d	Effective rate	Not reported
Daimeizhu (2014)	45 (23/22)	45 (25/20)	67.6 ± 6.4/69.5 ± 9.3	Random number table	EA plus Chinese medicine	Chinese medicine	Per day	16.75 ± 1.23/16.49 ± 1.36	Hubei	NA	4 w	PSQI; effective rate	Not reported
Liyuan (2022)	31 (16/15)	31 (17/14)	73.2 ± 1.7/73.4 ± 1.5	Not reported	EA plus Chinese medicine	Chinese medicine	1 time, 2 d	16.3 ± 2.4/16.7 ± 2.3	Guangdong	NA	3 w	PSQI; effective rate	Not reported
Maxinyu (2021)	50 (26/24)	50 (23/27)	71.2 ± 7.9/70.9 ± 8.5	Random toss	AC plus Chinese medicine	Chinese medicine	1 time, 2 d	13.27 ± 2.39/13.25 ± 2.41	Beijing	NA	4 w	Effective rate	Not reported
Niuqiyun (2021)	30 (18/12)	30 (20/10)	84.64 ± 2.67/84.57 ± 2.61	Not reported	AC plus estazolam	Estazolam	Per day	16.09 ± 1.15/16.00 ± 1.13)	Henan	NA	30 d	PSQI	Not reported
Wangjie (2020)	80 (46/34)	80 (39/41)	64.03 ± 2.87/64.93 ± 3.28	Not reported	AC plus psychological intervention	Psychological intervention	Per day	13.06 ± 2.57/12.81 ± 2.77	Shandong	NA	40 d	PSQI	Not reported
Wangyuliang (2021)	30 (14/16)	30 (12/18)	63.39 ± 4.732/64.38 ± 5.342	Random number table	AC plus Chinese medicine	Chinese medicine	2 times per day	14.34 ± 1.753/14.64 ± 1.729	Beijing	NA	18 d	PSQI; effective rate	Not reported
Xuyanjie (2018)	43 (13/30)	43 (12/31)	73.4 ± 11.6/74.5 ± 12.1	Random number table	AC plus cognitive behavior	Cognitive behavior	2 times, 1 wk	NA	Beijing	NA	8 w	Effective rate	Not reported
Yeshulan (2007)	30 (13/17)	28 (10/18)	68/65	Not reported	AC plus Chinese medicine	Chinese medicine	Per day	NA	Beijing	NA	30 d	Effective rate	Not reported
Liuli (2019)	39 (17/22)	39 (19/20)	75.20 ± 4.38	75.18 ± 4.32	AC plus estazolam and oryzanol	Estazolam and oryzanol	5 times, 1 wk	NA	Yunnan	NA	4 w	PSQI; effective rate	Not reported

PSQI = Pittsburgh Sleep Quality Index.

### 3.3. Study quality

**Figure 2.**
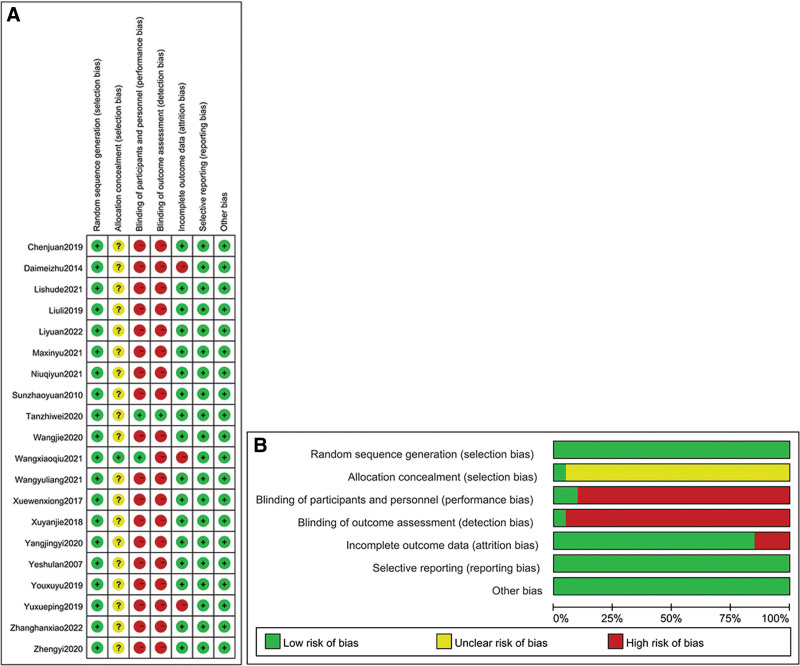
(A, B) The methodological quality of the included studies.

Of the 20 studies, 9 studies^[1,4,6,7,9,11,15,16]^ were randomized into groups using the random number table method, 1 study^[13]^ was randomized into groups through random toss, 1 study^[2]^ was randomized according to the order of treatment, and 9 studies^[3,5,8,10,12,14,17,18]^ did not mention the specific randomization method. Of the 20 studies, 1 study^[6]^ used allocation concealment, whereas 19 studies^[1–5,7–18]^ did not mention allocation concealment. Owing to the specificity of acupuncture operation, the studies displayed a high risk of bias in terms of blinding of participants and personnel: 1 study^[5]^ used the double-blinding method, 1 study^[6]^ used the single-blinding method, and 18 studies^[1–4,6–18]^ did not mention the blinding method used. In addition, 1 study^[6]^ blinded the result evaluation, and 19 studies^[1–5,7–18]^ did not mention whether the result evaluation was blinded. All of the 20 studies reported outcomes, with 3 studies^[1,6,11]^ demonstrating attrition bias. The methodological quality of specific studies is shown in Figure 2.

### 3.4. Meta-analysis findings

#### 3.4.1. Rate of improvement.

**Figure 3.**
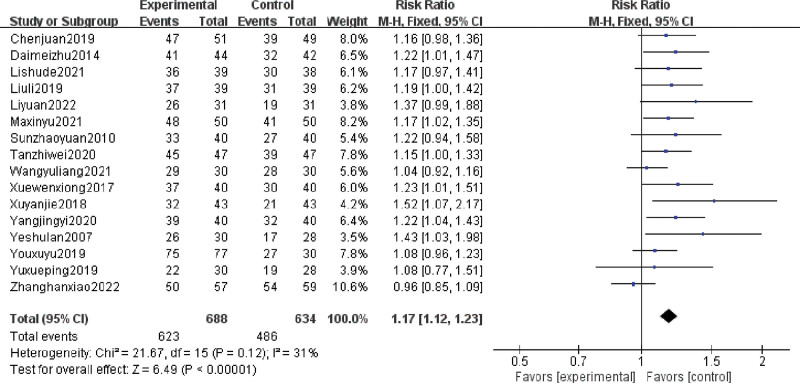
Forest plot for the rate of improvement. CI = confidence interval, Events = the effective number of patients, fixed = fixed effects model, M-H = Mantel–Haenszel method, total = the count of patients, weight = the credibility of the test.

A total of 16 RCTs reported the rate of improvement in sleep quality. The rate of improvement was significantly better in patients treated with acupuncture or moxibustion therapy than in those treated with other therapies (RR = 1.17; 95% CI, 1.12–1.23; Z = 6.49; *P *< .00001). No heterogeneity was observed among studies (Chi-square = 21.67; df = 15 [*P *= .12]; I^2^ = 31%) (Fig. 3).

**Figure 4.**
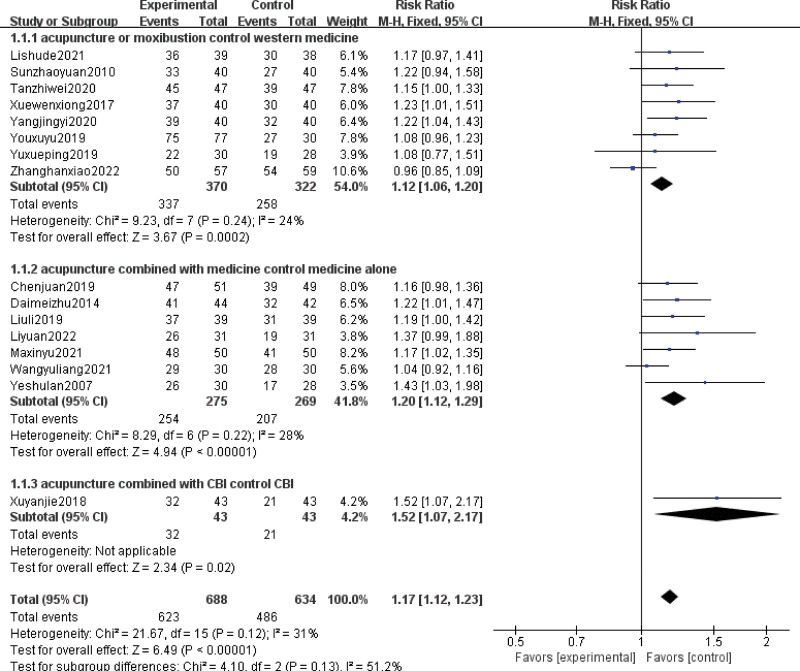
Subgroup analysis of the rate of improvement.

In the subgroup analysis, 8 studies reported the comparison between acupuncture or moxibustion alone and western drugs, 7 studies reported the comparison between the combination of acupuncture plus drugs and drugs alone, and 1 study reported the comparison between the combination of acupuncture plus other therapies (cognitive behavior intervention, CBT-I) and CBT-I alone. The rate of improvement of acupuncture or moxibustion alone was significantly better than that of western drugs (RR = 1.12; 95% CI, 1.06–1.20; Z = 3.67; *P *= .0002; heterogeneity test: Chi-square = 9.23, df = 7, *P *= .24, I^2^ = 24%). The rate of improvement of acupuncture combined with drugs was better than that of the drugs alone (RR = 1.20; 95% CI, 1.12–1.29; , Z = 4.94; *P *< .00001; heterogeneity test: Chi-square = 8.29, df = 6, *P *= .22, I^2^ = 28%). The rate of improvement of acupuncture combined with CBT-I was significantly better than that of CBT-I alone (RR = 1.52; 95% CI, 1.07–2.17; Z = 2.34; *P *= .02; heterogeneity was not assessed because there was only 1 study) (Fig. 4).

#### 3.4.2. PSQI score.

**Figure 5.**
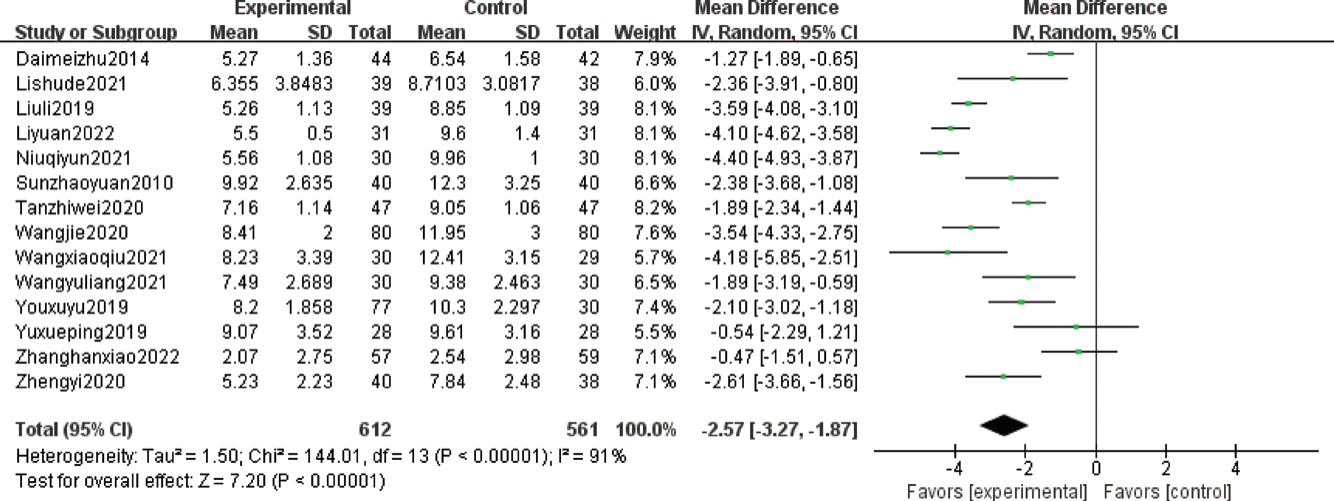
Forest plot of PSQI scores. CI = confidence interval, Mean = the average of outcomes, PSQI = Pittsburgh Sleep Quality Index, random = random effects model, SD = standard deviation, total = the count of patients, weight = the credibility of the test, IV = variance methods.

A total of 14 RCTs reported the use of PSQI for assessing sleep quality. Sleep quality was significantly better in patients treated with acupuncture or moxibustion therapy than in those treated with other therapies (MD = −2.57; 95% CI, −3.27 to −1.87; Z = 7.20; *P* < .00001). Heterogeneity was observed among studies (Chi-square = 144.01; df = 13 [*P* < .0001]; I^2^ = 91%) (Fig. 5). The results of sensitivity analysis were similar to and not significantly different from the above mentioned results.

**Figure 6.**
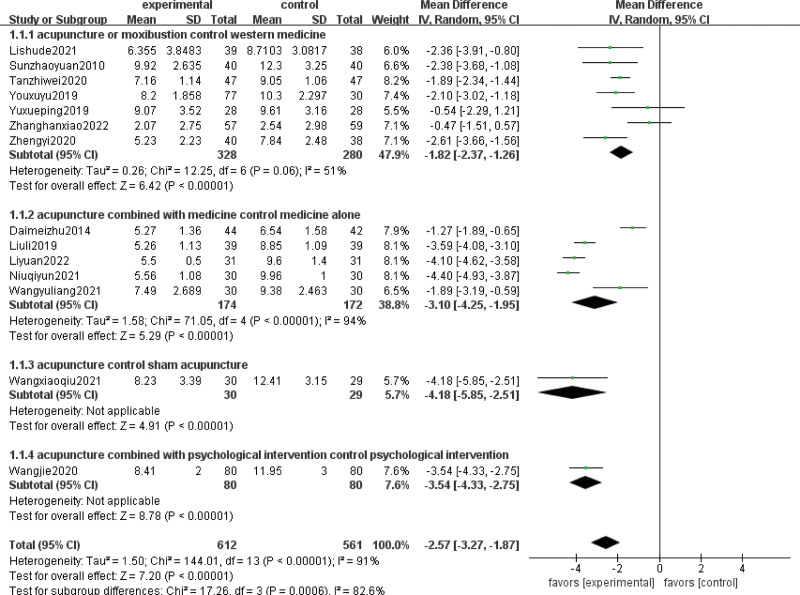
Subgroup analysis of PSQI scores. PSQI = Pittsburgh Sleep Quality Index.

In the subgroup analysis, 7 RCTs reported the comparison between acupuncture or moxibustion alone and western drugs, 5 RCTs reported the comparison between acupuncture combined with drugs and drugs alone, 1 RCT reported the comparison between acupuncture and sham acupuncture, and 1 RCT reported the comparison between acupuncture combined with psychological intervention and psychological intervention alone. The PSQI scores of patients treated with acupuncture or moxibustion alone were better than those of patients treated with western drugs; a random effects model was used for analysis (MD = −1.82; 95% CI, −2.37 to −1.26; Z = 6.42; *P *< .00001; heterogeneity test: Chi-square = 12.25, df = 6 [*P *= .06], I^2^ = 51%). Acupuncture combined with drugs was more effective than the drugs alone in improving PSQI scores (MD = −3.10; 95% CI, −4.25 to −1.95; Z = 5.29; *P* < .00001; heterogeneity test: Chi-square = 71.05, df = 4 [*P* < .00001], I^2^ = 94%). Acupuncture was significantly more effective than sham acupuncture (MD = −4.18; 95% CI, −5.85 to −2.51; Z = 4.91; *P* < .00001) and psychological intervention (MD = −3.54; 95% CI, −4.33 to −2.75; Z = 8.78; *P* < .00001) in improving PSQI scores. Heterogeneity was not assessed because there was only 1 study reporting the comparison between acupuncture and sham acupuncture or psychological intervention (Fig. 6).

#### 3.4.3. Publication bias.

**Figure 7.**
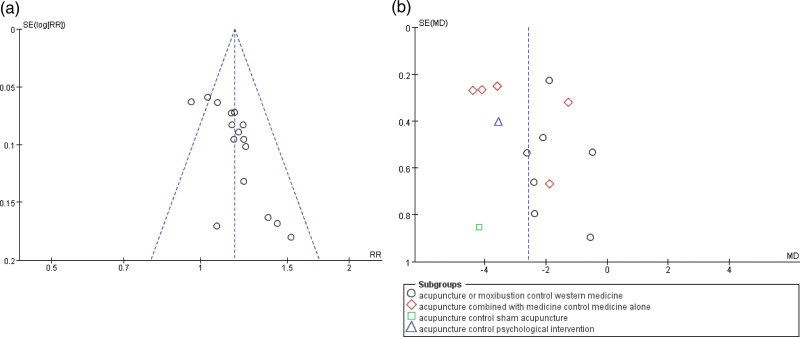
(A) Funnel plot of the rate of improvement. (B) Funnel plot of PSQI scores. PSQI = Pittsburgh Sleep Quality Index.

**Figure 8.**
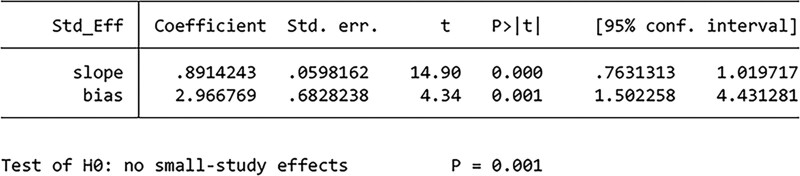
Egger test results of the rate of improvement.

**Figure 9.**
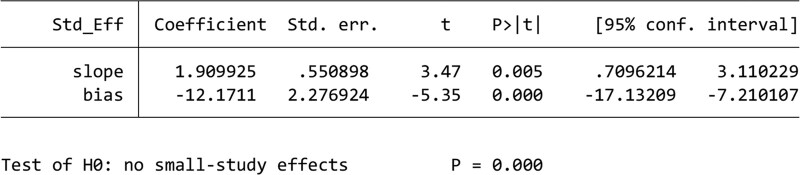
Results of Egger test of PSQI scores. PSQI = Pittsburgh Sleep Quality Index.

Funnel plots constructed to demonstrate the rate of improvement (Fig. 7A) and PSQI scores (Fig. 7B) indicated the presence of publication bias. In addition, Egger test performed to compare the rate of improvement (Fig. 8) (*P *= .001) and PSQI scores (Fig. 9) (*P *= 0) among studies indicated the presence of publication bias. Publication bias may be attributed to the withholding of negative results from publication.

## 4. Discussion

Human sleep is a complex physiological process that is closely related to the release of neurotransmitters and metabolic activity.^[29]^ According to traditional Chinese medicine, yang cannot enter yin owing to insufficient qi and blood; therefore, insomnia often occurs in the elderly population. Studies have demonstrated that acupuncture or moxibustion can stimulate acupoints to increase the concentration of β-endorphin, endogenous melatonin, NO, or γ-aminobutyric acid, thereby improving sleep quality.^[30–32]^

In this systematic review, we focused on primary insomnia. Studies involving other conditions or diseases, such as depression, stroke, and cancer, were excluded. This systematic review and meta-analysis aimed to assess the effectiveness of acupuncture or moxibustion in treating senile insomnia. The results of 20 RCTs involving 1677 patients with senile insomnia showed that acupuncture or moxibustion treatment was more effective in ameliorating insomnia and improving PSQI scores than conventional medicine, CBT-I, or psychological intervention. Of the 20 included studies, 9 studies reported the comparison between acupuncture or moxibustion alone and western drugs, 8 studies reported the comparison between acupuncture and drugs, 1 study reported the comparison between acupuncture and sham acupuncture, and 2 studies reported the comparison between acupuncture combined other therapies (CBT-I or psychological intervention) and other therapies alone.

However, subgroup analysis was performed owing to differences in treatment methods. Compared with drugs alone, acupuncture or moxibustion either alone or combined with drugs significantly improved sleep quality and PSQI scores. But because only 3 studies respectively reported the use of sham acupuncture, CBT-I, or psychological therapy in the control group, their results and conclusions were unconvincing.

PSQI is widely used to assess sleep quality. Compared with drugs, acupuncture and moxibustion were more effective in improving PSQI scores. However, these scores were graded by patients and hence have strong subjectivity. This discrepancy may have resulted in bias, which explains the heterogeneity observed in the rate of improvement among studies.

However, this meta-analysis has some limitations. First, the heterogeneity of PSQI scores was high; therefore, a definite conclusion could not be drawn. However, WMDs of statistical significance have been provided in the Forest plot of individual studies. Second, the methodological quality of the included studies was low. An unclear or high risk of bias was observed in terms of blinding, random sequence generation, and allocation concealment in the included studies. Therefore, it is necessary to record all relevant information to minimize performance and assessment bias and improve the quality of study design in the future. Third, the criteria for assessing the rate of improvement were different among the included studies, which increased bias in the pooled rate of improvement in sleep quality to some extent. However, owing to an insufficient number of studies focusing on senile insomnia, studies involving the rate of improvement were included. A standardized criterion should be provided for more robust conclusions regarding the effectiveness of each treatment method in relieving the symptoms of insomnia. Finally, the participants in the included studies were selected from a single center in China, which may result in a lack of representativeness. Moreover, owing to the lack of follow-up data, the long-term effects of acupuncture could not be examined. These limitations should be addressed in future studies.

## 5. Conclusion

Acupuncture or moxibustion alone or combination with drugs may be more effective in treating senile insomnia than drugs alone. Since there was only one study in which the control group was sham acupuncture, CBT-I, or psychological intervention, the result is unconvincing that acupuncture is better than the above therapies in the treatment of senile insomnia. In addition, acupuncture or moxibustion appears to have long-term benefits. However, further well-designed studies are warranted to verify these findings.

## Author contributions

**Conceptualization:** Wenjiao Hu, Hao Zhou, Yue Zeng, Qian Zeng, Zubo Huang, Chao Wang.

**Data curation:** Wenjiao Hu, Hao Zhou.

**Formal analysis:** Wenjiao Hu, Hao Zhou, Yue Zeng, Qian Zeng.

**Funding acquisition:** Chao Wang.

**Methodology:** Wenjiao Hu, Hao Zhou, Yue Zeng, Qian Zeng, Zubo Huang.

**Resources:** Wenjiao Hu, Hao Zhou, Yue Zeng.

**Software:** Wenjiao Hu, Hao Zhou, Qian Zeng.

**Supervision:** Wenjiao Hu, Hao Zhou, Yue Zeng, Qian Zeng, Zubo Huang.

**Visualization:** Wenjiao Hu, Hao Zhou, Yue Zeng, Qian Zeng, Zubo Huang.

**Validation:** Hao Zhou.

**Writing – original draft:** Wenjiao Hu, Hao Zhou.

**Writing – review & editing:** Wenjiao Hu, Hao Zhou, Zubo Huang, Chao Wang.

## Supplementary Material





## References

[1] Farazdaq H Andrades M Nanji K. Insomnia and its correlates among elderly patients presenting to family medicine clinics at an academic center. Malays Fam Physician. 2018;13:12–9. 30800228 PMC6382090

[2] Leung A Leung A Wong A. Sleep terrors: an updated review. Curr Pediatr Rev. 2020;16:176–82. 31612833 10.2174/1573396315666191014152136 PMC8193803

[3] Patel D Steinberg J Patel P. Insomnia in the elderly: a review. J Clin Sleep Med. 2018;14:1017–24. 29852897 10.5664/jcsm.7172 PMC5991956

[4] Gooneratne NS Vitiello MV. Sleep in older adults: normative changes, sleep disorders, and treatment options. Clin Geriatr Med. 2014;30:591–627. 25037297 10.1016/j.cger.2014.04.007 PMC4656195

[5] Shekleton JA Rogers NL Rajaratnam SM. Searching for the daytime impairments of primary insomnia. Sleep Med Rev. 2010;14:47–60. 19963414 10.1016/j.smrv.2009.06.001

[6] Wickwire EM Amari DT Juday TR. Cardiac events and economic burden among patients with hypertension and treated insomnia in the USA. Future Cardiol. 2022;18:731–41. 35787013 10.2217/fca-2022-0009

[7] Blake MJ Trinder JA Allen NB. Mechanisms underlying the association between insomnia, anxiety, and depression in adolescence: Implications for behavioral sleep interventions. Clin Psychol Rev. 2018;63:25–40. 29879564 10.1016/j.cpr.2018.05.006

[8] Bollu PC Kaur H. Sleep medicine: insomnia and sleep. Mo Med. 2019;116:68–75. 30862990 PMC6390785

[9] Yu XP Gao QC. Clinical study on the effect of acupuncture on sleep quality and cognitive function in senile patients with primary insomnia. Jiangsu J Tradit Chin Med. 2019;51:62–4.

[10] Sun ZY. Treatment of 40 cases of insomnia by acupuncture at Taixi Sanyinjiao Yongquan Acupoint. Shaanxi J Tradit Chin Med. 2010;31:731–2.

[11] Li SD. Clinical observation on the treatment of senile insomnia of heart-kidney dissonant type with ‘Regulating Qi and Regressing Yuan. Fujian Univ Chin Med. 2021.

[12] Tan ZW. Yang hidden in Yin’ acupuncture method for the treatment of senile insomnia in the clinical observation. J Massage Rehabil Med. 2020;11:25–6.

[13] Wang XQ Qin S Wu WZ. Treatment of senile insomnia by electroacupuncture and its effect on serum melatonin and dopamin. Chin J Acupunct Moxibustion. 2021;41:501–4. 10.13703/j.0255-2930.20200404-k0001 34002562

[14] You XY Chen XK Wang CY. Clinical observation on the treatment of insomnia in elderly patients with “awakening brain and opening body” acupuncture. World Latest Med Inf Abstr. 2019;19:144–5.

[15] Yang JY Ma NQ Zhang JJ. Observation of clinical effect of acupuncture and moxibustion on senile insomnia. Special Health. 2020:56.

[16] Zhang HX Guo W Lin WF. Clinical study on the treatment of senile insomnia by “Regulating Qi and Regressing Yuan” acupuncture and moxibustion. Health World. 2022:51–2.

[17] Chen J Zhou LK Zhan D. Analysis on the clinical effect of Dong Shi Unusal Acupoint Combined with Yangxin Anshen Expectorant Decoction on the elderly patients with phlegm-dampness inherent insomnia. Hebei J Tradit Chin Med. 2019;34:37–40.

[18] Dai MZ Zhang XF Guo W. Clinical observation on senile insomnia treated by acupuncture Sishencong acupoint combined with Shumian Capsule. J Hubei Univ Tradit Chin Med. 2014;16:103–5.

[19] Ma XY Wang LP Liu HL. Nourishing Yin and Clearing Heat Decoction combined with acupuncture and moxibustion treat elderly insomnia with Yin deficiency and blood sufficiency and restlessness. Liaoning J Tradit Chin Med. 2021;48:207–10.

[20] Niu QY Miao ZG. Clinical observation of acupuncture on infusion acupoints on the back in the treatment of deficiency of heart and spleen in senile insomnia. Chin J Gerontol. 2021;41:5024–7.

[21] Wang J Liu Y Zhang YL. Therapeutic effect of acupuncture and moxibustion combined with psychological counseling on insomnia in the elderly. Chin J Gerontol. 2020;40:2598–600.

[22] Wang YL Zhang R Wang CX. Observation on the curative effect of acupuncture Dong Shi Unusal Acupoint combined with Sini Powder in the treatment of senile insomnia with liver depression and spleen deficiency. Chin J Geriatr Med. 2021;19:17–9.

[23] Xu YJ Wang R Yang ZQ. Effect of single therapy and combination of two therapies on chronic insomnia in the elderly. Med J Armed Police Force. 2018;29:1125–8.

[24] Ye SL. Treatment of 58 cases of intractable insomnia with acupuncture and medicine. Clin J Acupunct Moxibustion. 2007:16–7.

[25] Liu L. Evaluation of short-term efficacy of acupuncture in treating senile insomnia. Family Medicine. Chinese J Med. 2019:68.

[26] Buysse DJ Reynolds CR Monk TH. The Pittsburgh sleep quality index: a new instrument for psychiatric practice and research. Psychiatry Res. 1989;28:193–213. 2748771 10.1016/0165-1781(89)90047-4

[27] Barcot O Ivanda M Buljan I. Enhanced access to recommendations from the Cochrane Handbook for improving authors’ judgments about risk of bias: a randomized controlled trial. Res Synth Methods. 2021;12:618–29. 34050603 10.1002/jrsm.1499

[28] Ioannidis JP Trikalinos TA. The appropriateness of asymmetry tests for publication bias in meta-analyses: a large survey. CMAJ. 2007;176:1091–6. 17420491 10.1503/cmaj.060410 PMC1839799

[29] Proserpio P Marra S Campana C. Insomnia and menopause: a narrative review on mechanisms and treatments. Climacteric. 2020;23:539–49. 32880197 10.1080/13697137.2020.1799973

[30] Cheng CH Yi PL Lin JG. Endogenous opiates in the nucleus tractus solitarius mediate electroacupuncture-induced sleep activities in rats. Evid Based Complement Alternat Med. 2011;2011:159209. 19729491 10.1093/ecam/nep132 PMC3094708

[31] Fu LW Longhurst JC. Electroacupuncture modulates vlPAG release of GABA through presynaptic cannabinoid CB1 receptors. J Appl Physiol (1985). 2009;106:1800–9. 19359606 10.1152/japplphysiol.91648.2008 PMC2692780

[32] Li S Chen K Wu Y. Effects of warm needling at zusanli (ST 36) on NO and IL-2 levels in the middle-aged and old people. J Tradit Chin Med. 2003;23:127–8. 12875077

