# Supplementary material for: Efficacy of acupuncture or moxibustion in treating senile insomnia compared with a control group: A systematic review and meta-analysis
Source: Medicine (Baltimore). 2023 Oct 20;102(42):e34842. doi: 10.1097/MD.0000000000034842 (PMC10589601; doi:10.1097/MD.0000000000034842)
Supplement: Supplementary file 1 [file medi-102-e34842-s001.doc]

**Research strategy：**

(((((("Sleep Initiation and Maintenance Disorders"[Mesh]) OR (((((((((((((((((((((((((((Disorders of Initiating[Title/Abstract] OR Maintaining Sleep[Title/Abstract])OR ((DIMS (Disorders of Initiating[Title/Abstract] OR Maintaining Sleep[Title/Abstract])) OR (Early Awakening[Title/Abstract])) OR (Awakening, Early[Title/Abstract])) OR (Nonorganic Insomnia[Title/Abstract])) OR (Insomnia, Nonorganic[Title/Abstract])) OR (Primary Insomnia[Title/Abstract])) OR ((Insomnia, Primary[Title/Abstract])) OR (Transient Insomnia[Title/Abstract])) OR (Insomnia, Transient[Title/Abstract])) OR (Rebound Insomnia[Title/Abstract])) OR (Insomnia, Rebound[Title/Abstract])) OR (Secondary Insomnia[Title/Abstract])) OR (Insomnia, Secondary[Title/Abstract])) OR (Sleep Initiation Dysfunction[Title/Abstract])) OR (Dysfunction, Sleep Initiation[Title/Abstract])) OR (Dysfunctions, Sleep Initiation[Title/Abstract])) OR (Sleep Initiation Dysfunctions[Title/Abstract])) OR (Sleeplessness[Title/Abstract])) OR (Insomnia Disorder[Title/Abstract])) OR (Insomnia Disorders[Title/Abstract])) OR (Insomnia[Title/Abstract])) OR (Insomnias[Title/Abstract])) OR (Chronic Insomnia[Title/Abstract])) OR (Insomnia, Chronic[Title/Abstract])) OR (Psychophysiological Insomnia[Title/Abstract])) OR (Insomnia, Psychophysiological[Title/Abstract]))) OR (pervigilium[Title/Abstract]) OR (agrypnia[Title/Abstract]))) OR (((((sleeplessness[Title/Abstract]) OR (hyposomnia[Title/Abstract])) OR (sleeplessness[Title/Abstract])) OR (sleep disorder[Title/Abstract])) OR (dyssomnia[Title/Abstract]))) AND ((senile[Title/Abstract]) OR (elder[Title/Abstract]))) AND ((((((("Acupuncture, Ear"[Mesh]) OR ( "Acupuncture Therapy"[Mesh] OR "Acupuncture"[Mesh] )) OR ("Moxibustion"[Mesh] Sort by: Most Recent)) OR (moxibustion[Title/Abstract])) OR (((((( "Acupuncture Therapy"[Mesh] OR "Acupuncture"[Mesh] ))) OR ("Moxibustion"[Mesh])) OR (moxibustion[Title/Abstract]))) OR (("Electroacupuncture"[Mesh]) OR (Electroacupuncture[Title/Abstract])))) AND (randomized controlled trial[Publication Type] OR randomized[Title/Abstract] OR placebo[Title/Abstract])
